# miR‐31‐5p suppresses myocardial hypertrophy by targeting Nfatc2ip

**DOI:** 10.1111/jcmm.18413

**Published:** 2024-06-19

**Authors:** Lamei Zhao, Xiaotao Qian, Zhenxing Ren, Ailing Wang

**Affiliations:** ^1^ Department of Cardiology 1st Affiliated Hospital of Anhui Medical University Hefei Anhui China; ^2^ Department of Oncology, Hefei Cancer Hospital Chinese Academy of Sciences Hefei Anhui China; ^3^ Department of Anatomy, The Research Center of Basic Integrative Medicine Guangzhou University of Traditional Chinese Medicine Guangzhou Guangdong China

**Keywords:** miR‐31‐5p, myocardial hypertrophy, Nfatc2ip, β‐Mhc

## Abstract

Cardiac hypertrophy, worldwide known as an adaptive functional compensatory state of myocardial stress, is mainly believed to proceed to severe heart diseases, even to sudden death. Emerging studies have explored the microRNA alteration during hypertrophy. However, the mechanisms of microRNAs involved in cardiac hypertrophy are still uncertain. We studied young rats to establish abdominal aorta coarctation (AAC) for 4 weeks. With the significant downregulated cardiac function and upregulated hypertrophic biomarkers, AAC‐induced rats showed enlarged myocardiocytes and alterations in microRNAs, especially downregulated miR‐31‐5p. miR‐31‐5p targets the 3′UTR of Nfatc2ip and inhibits myocardial hypertrophy in vitro and in vivo. Furthermore, we verified that Nfatc2ip is necessary and sufficient for cardiac hypertrophy in neonatal rat cardiomyocytes. Moreover, we found miR‐31‐5p inhibited the colocalization of Nfatc2ip and hypertrophic gene β‐Mhc. Luciferase assay and ChiP‐qPCR test demonstrated that Nfatc2ip binded to the core‐promoter of β‐Mhc and enhanced its transcriptional activity. Above all, our study found a new pathway, mir‐31‐5p/Nfatc2ip/β‐Mhc, which is involved in cardiac hypertrophy, suggesting a potential target for intervention of cardiac hypertrophy.

## 1 INTRODUCTION

Worldwide, cardiac hypertrophy is thought to be an adaptive functional compensatory state of myocardial stress (e.g. persistent and persistence of pressure overload). It is now believed to proceed to heart failure, which affects more than 26 million people worldwide and is well known as a risk of sudden death.^1^ The mechanism of pressure overload‐induced cardiac hypertrophy has been characterized as increased left ventricular (LV) wall thickness and impaired cardiac function in a process called concentric hypertrophy.^2^ Moreover, whole organ remodelling and hypertrophy are usually associated with similar changes at the myocardiocyte level, increased size and increased biomarker genes such as ANP, BNP and β‐Mhc.^3^, ^4^

Recently, accumulative studies have demonstrated that transcriptional and post‐transcriptional regulations are involved in pressure overload‐induced remodelling. The enhanced transcriptional regulations such as histone deacetylases mediated chromatin alteration or the calcineurin‐nuclear factor of activated T cell (NFAT) signalling, as well as the activated transcriptional factors including CREB binding protein, GATA4 and MEF2, respond to pathological stimuli.^4^, ^5^, ^6^, ^7^, ^8^ Nfatc2ip is one of the NFAT families, which has been thought to promote cardiac hypertrophy and pathological remodelling,^9^ and disrupt NFAT signalling to attenuate cardiac hypertrophy.^10^ Interestingly, one published study has observed that Nfatc2ip significantly upregulated in the third month after ST‐elevation myocardial infarction,^11^ which is close to LV hypertrophy.^12^

Increasing evidence reported that microRNAs (miRNAs), mainly involved in post‐transcriptional regulation of gene expression, play crucial roles in the regulatory network in cardiovascular disease, including cardiac hypertrophy. Two categories defined as pro‐hypertrophic and anti‐hypertrophic microRNAs are involved in cardiac hypertrophy. MicroRNAs, including miR‐155, miR‐22, miR‐217 etc., are reported to promote cardiac hypertrophic processes,^7^, ^13^, ^14^, ^15^, ^16^, ^17^ whereas miR‐1, miR‐133a, miR‐142‐3p, etc., are found to attenuate cardiac hypertrophy.^18^, ^19^ Though the mechanism of microRNAs involved in cardiomyocyte remodelling has been researched, the mechanism of microRNA involved in overload‐induced cardiac hypertrophy in the early stage remains uncertain.

In this study, microarrays and qPCR revealed that miR‐31‐5p was dominantly increased in 4‐week abdominal aorta coarctation (AAC)‐inducted rats. Then, we verified that miR‐31‐5p suppresses cardiac hypertrophy in vivo and in vitro. Nfatc2ip was the target of miR‐31‐5p, confirmed by dual‐luciferase assay. Moreover, overexpressed Nfatc2ip induced myocardial hypertrophy in the primary cell, which is rescued by si‐Nfatc2ip RNA. Further, we found that Nfatc2ip activates *β‐Mhc* transcription by targeting the core‐promoter of *β*‐Mhc, and miR‐31‐5p inhibits Nfatc2ip‐*β‐Mhc* signalling to prevent cardiac hypertrophy.

## 2 MATERIALS AND METHODS

### 2.1 Construction of a rat model of pressure‐overload myocardial hypertrophy

Male Sprague–Dawley (SD) rats (80–100 g) were provided by Shanghai SLAC Laboratory Animal Co., Ltd. Rats were housed with free access to food and water at a constant temperature of 24 ± 1°C, a humidity of 55 ± 5% and a 12 h light/dark cycle, with adequate food and water.

Rats' cardiac hypertrophy model (*n* = 8) was induced by AAC for 4 weeks. The abdominal aorta was occluded at the suprarenal level using a 2‐0 silk suture over a blunt 24‐G needle (the external diameter was 0.55 mm).^20^ Rats were anaesthetised using 2% isoflurane (R510‐22‐16, RWD Life Science) and oxygen with a flow rate of 0.5 L/min and maintained with 1.5% isoflurane with an airflow of 0.3 L/min in an induction chamber connected with an animal anaesthesia machine. The sham groups (*n* = 8) underwent similar surgery without placement of the aortic band.

To evaluate the miR‐31‐5p effect on cardiac hypertrophy, the rats' AAC model was performed on Day 7, and the injection of miR‐31‐5p agomir, antagomir and the scramble was performed on Days 10 and 21 respectively. The sham group was processed in the same way with the same value of saline sodium injected.

The rats were anaesthetized with pentobarbital sodium (30 mg/kg, intraperitoneal injection), killed, blood taken from the abdominal aorta and tissues harvested.

### 2.2 Agomir and antagomir were administered by tail vein injection

The miR‐31‐5p Agomir (5 mg/kg, RiboBio Co., Ltd.), antagomir (5 mg/kg), and Scramble (5 mg/kg) were injected via tail vein separately on Day 10 (the third day after AAC modelling) and administered twice using the exact dosage at Day 21. The sham group and AAC group rats were injected with the same volume of normal saline.

### 2.3 MicroRNA microarray assay

The heart tissues were taken for the miRNA microarray assay, which was performed by Guangzhou RiboBio Co., Ltd. The experiment included pre‐hybridization, hybridization, washing and imaging. GustomArray™ microarray was assembled with a hybridization cap and clips. The microarray was rinsed to decrease the unspecific hybridization background, was covered with the imaging solution and was loaded into the Genepix 400B microarray scanner to scan.

### 2.4 Bioinformatics analysis

miRBase, microRNA.org and miRcode bioinformatics were used to predict potential target genes of miR‐31‐5p.

### 2.5 Echocardiography

Rats were lightly anaesthetised with pentobarbital sodium (30 mg/kg, intraperitoneal injection) and placed on a platform. Cardiac anatomical and functional parameters were evaluated by two‐dimensional transthoracic echocardiography using a visual sonic ultrasound system (vevo2100). Averaged LV diastolic and systolic anterior wall thickness (LVAWd, LVAWs), LV diastolic and systolic posterior wall thickness (LVPWd, LVPWs), and LV diastolic and systolic internal dimensions (LVIDd, LVIDs) were measured. LV fractional shortening (FS) [(LVIDd − LVIDs)/LVIDd] and LV mass (LV Mass) = 1.053 × [(LVID; d + LVPW; d + LVAW; d) 3 − LVID; d3] were calculated from the M‐mode measurements. LV ejection fraction (EF) was calculated from the LV cross‐sectional area (2‐D short‐axis view) using the equation LV %EF = (LV Vol; d‐LV Vol; s)/LV Vol; d × 100%.^8^

### 2.6 Neonatal rat cardiomyocytes and H9C2 cell culture

Neonatal rat cardiomyocytes (NRCMs) were isolated from neonatal Sprague–Dawley rat pups (newborn in 24 h) using the neonatal cardiomyocyte isolation system (Worthington Biochemical Co, Lakewood, NJ). Furthermore, cells were plated into a 6‐well plate at a density of 2.5 × 10^5^ cells on 1% gelatin‐coated plates and cultured in Dulbecco's modified Eagle's medium (DMEM) supplemented with 10% (v/v) fetal bovine serum (Gibco) and 1% antibiotics (penicillin and streptomycin, Gibco) at 37°C in humidified air with 5% CO_2_.

### 2.7 Cell size measurement

The cell size was detected as described in the references. Moreover, tissues and cells were fixed with 4% paraformaldehyde (PFA) for 48 h and 30 min, respectively, followed by staining with wheat germ agglutinin (WGA, 1:500L4895; sigma) for tissues or actin stained with Phalloidin‐iFluor 488 (1:1000, ab176753, Abcam) for cells. The nucleaus were stained with 4′,6‐diamidino‐2‐phenylindole (DAPI, 1:1000, D8200, Solarbio) or propidium iodide (PI, 1:50, ab14083, Abcam) combined using RNaseA (1:100, ab300192, Abcam) for 30 min at 37°C. The images were captured using a confocal microscope (LSM 880, Zeiss), and the area of the cells was measured by ImageJ.

### 2.8 Western blot analysis

Proteins were extracted from the frozen fresh heart tissue by RIPA lysis buffer (89901#, Thermo) and added with a cocktail (1:100, B14001, Bimake) to prevent protein enzyme. The supernatant of each group was obtained by centrifugation at 12,000×*g* at 4°C for 20 min and quantified with the BCA Protein assay (23225#, Thermo). The protein extracts added with loading buffer were boiled for 10 min, and separated by 10% SDS‐PAGE, electrophoretically transferred to microporous polyvinylidene difluoride membranes (0.22 μm, Millipore, Germany). The blot was probed with antibodies including Nfatc2ip (1:1000, sc‐377461, Santa Cruz), Anp (1:5000, ab225844, Abcam) and β‐Mhc (1:1000, sc‐53090, Santa Cruz), horseradish‐peroxidase (HRP)‐labelled GAPDH (1:10, 000, HRP‐60004, Proteintech) for 120 min followed by HRP‐labelled secondary antibodies (anti mouse:1:10, 000, ab97040, Abcam; anti‐rabbit: 1:10, 000, ab6721, Abcam) at room temperature for 60 min. Based on the manufacturer's protocol, bands were visualized using a chemiluminescence kit (WBKLS0500, Millipore).

### 2.9 Immunohistochemical staining

Immunofluorescence was conducted following a two‐step protocol in which the sections or wells were first incubated with Nfatc2ip (1:500, sc‐377461, Santa Cruz). The secondary antibodies used were Alexfluor488 (1:1000, ab150113, Abcam); subsequently, the sections were incubated with DAPI. As Nfatc2ip and β‐Mhc are from the same species (mouse), which may affect their co‐staining, we used the CF‐647 dye to conjugate to β‐Mhc antibody. After staining with Nfatc2ip and Alexfluor 488, and washing three times, β‐Mhc‐labelled CF647 (1:100) were incubated for 1 h at room temperature followed by incubation with DAPI. The images were captured using a confocal microscope (LSM 880, Zeiss), and the area of the cells was measured by ImageJ.

### 2.10 Real‐time quantity PCR analysis

The total RNA lysates were prepared using Trizol Reagent (139505, Life) for 30 min on ice and then isolated using Direct‐zolTM RNA MiniPrep Plus (ZRC200606, Zymo Research). The quantity and the quality of RNAs were analysed on a NanoDrop 2000 spectrophotometer (Thermo Fisher Scientific). cDNAs were prepared using Transcriptor First Strand cDNA Synthesis Kit (TAKARA), with a mix of dT primers for mRNA and stem‐loop primers (Sangong, Shanghai) for miRNA, at 50°C for 1 h. The qPCR was performed using SYBR Green master mix (TAKARA) with appropriate primers. Relative quantification of gene expression was conducted with the Bio‐Rad CFX96 (Bio‐Rad) and Real‐Time PCR System. Relative quantification was carried out with the 2^−∆∆Ct^ method. The GAPDH mRNA was used to control mRNA expression and U6 for miRNA. The primers are shown as follows:

**Table:** 

Natc2ip‐F	ACACTTCCAGCTTCGGAGGA
Natc2ip‐R	TGCTGCTTGGCTAATCCAGA
GAPDH‐F	GTGTCCAATGCAGCTTGAGG
GAPDH‐R	TCTGCCACCACCACAGACTT
microRNAs‐R	ATCCAGTGCAGGGTCCGAGG
mir‐31‐5p‐RT	GTCGTATCCAGTGCAGGGTCCGAGGTATTCGCACTGGATACGACCAGGGA
mir‐31‐5p‐F	GCGCGTGAGATGGCTCCCTG
mir‐20b‐3p‐RT	GTCGTATCCAGTGCAGGGTCCGAGGTATTCGCACTGGATACGACCCTGCA
mir‐20b‐3p‐F	CAAAGTGCTCATAGTGCAGG
let‐7a‐5p‐RT	GTCGTATCCAGTGCAGGGTCCGAGGTATTCGCACTGGATACGACGGAAAG
let‐7a‐5p‐F	CTATACAATCTACTGTCTTTCC
mir‐133‐5p‐RT	GTCGTATCCAGTGCAGGGTCCGAGGTATTCGCACTGGATACGAC
mir‐133‐5p‐F	CCAAGGTAAAATGGTCGA
mir‐26‐5p‐RT	GTCGTATCCAGTGCAGGGTCCGAGGTATTCGCACTGGATACGACTCGACC
mir‐26‐5p‐F	CGGATAGGACCTAATGAAC
mir‐488‐3p‐RT	GTCGTATCCAGTGCAGGGTCCGAGGTATTCGCACTGGATACGACGACCAA
mir‐488‐3p‐F	GAAAGGCTGTTTCTTGGTC
U6‐F	AGAGAAGATTAGCATGGCCCCTG
U6‐R	ATCCAGTGCAGGGTCCGAGG

### 2.11 Transfection of miR‐31‐5p mimics and inhibitors to NRCMs and H9C2 cells

The NRCMs were transfected with miR‐31‐5p mimics and inhibitors with Scramble as normal control using Lipofectamine RNAiMAX (13778075, Invitrogen) transfection reagent after plating for 18 h. After 24 h, NRCMs were treated with ANGII (500 nM) containing medium for another 24 h. NRCMs were harvested by fixing with 4% PFA or stored at −80°C.

H9C2 cells were plated into 6‐well cell culture clusters and transfected with 100 nM miR‐31‐5p mimics or inhibitors for 6 h according to the protocol of Lipofectamine 2000. The cells were collected after the terminal transfection for 24 h with 0.025% trypsin digestion and lysis with RIPA buffer for western blot analysis.

### 2.12 Nfatc2ip‐overexpressed H9C2 cell line

H9C2 cells were infected with lentivirus generated with the plasmid containing EF1a‐Nfatc2ip‐eGFP‐CMV‐puro and packaging plasmids (OBiO Technology Corp., Ltd., Shanghai). After 8h infection, 1 μg/mL puromycin (P8230, Solarbio) was added to the culturing medium to screen the positive cells for later use in the experiments.

### 2.13 Co‐transfection of plasmid into H9C2 cells

H9C2 cells at a density of 2 × 10^4^ cells per well were transfected using Lipofectamine 3000 (Invitrogen) with pisCHECK‐Nfatc2ip construct plasmid (200 ng) and miR‐31 mimic or inhibitor (100 nM) for 6–8 h in a 24‐well cell. They were then altered with fresh medium for another 24‐h culture. Thereafter, cells were collected and assayed with the Dual‐Luciferase Reporter Assay System to quantify the luminescent signal. In the cloning vector of pisCHECK‐Nfatc2ip, microRNA target sites were inserted after the Rellina luciferase (hRluc) region and Firefly luciferase (hluc) was taken as an internal reference.

We set up five groups as follows: Nfatc2ip^WT^+S, Nfatc2ip^WT^+M, Nfatc2ip^WT^+I, Nfatc2ip^Mut^+M and Nfatc2ip^Mut^+I. WT means wild type, Mut means Mutant type, S represents Scramble miRNA, M means miR‐31‐5p mimic and I means miR‐31‐5p inhibitor.

### 2.14 Chromatin immunoprecipitation

The nucleoprotein extracts were acquired using a kit (78833, Thermo). The chromatin was sheared with sonication equipment in an ice bath, with 70% adequate power, 5 s on and 10 s off, total of 50 cycles. Thereafter, the sheared DNA fragment was loaded onto 1% agarose gel with a running condition of 140 V, 40 min. The chromatin immunoprecipitation procedures were performed according to the protocol of the EZ‐Magna Chromatin immunoprecipitation kit (17‐10086; Millipore). The primers qPCR are as follows: β‐Mhc‐promoter‐R: GGGATCCGTCCCCAGCTCTG; β‐Mhc‐promoter‐R: GATGGTCTCCCACTGATACC.

### 2.15 Statistical analysis

All data in our study were expressed at mean ± standard deviation (SD). Analysis of variance was carried out with GraphPad prism9 (Version 9.3.0). Statistical comparison between the two groups was performed by Student's *t*‐test. Moreover, microRNAs were validated using multiple unpaired *t*‐tests with multiple comparisons as false discovery rate (FDR; two‐stage set‐up; desired FDR: 1%). For two groups, unpaired two‐tailed *t*‐tests were used, and for more than two groups, they were treated with one‐way ANOVA. The difference was considered statistically significant at *p < 0.05, **p < 0.01 and ***p < 0.001.

## 3 RESULTS

### 3.1 miR‐31‐5p is downregulated in AAC‐induced myocardial hypertrophy and dysfunction

**FIGURE 1:**
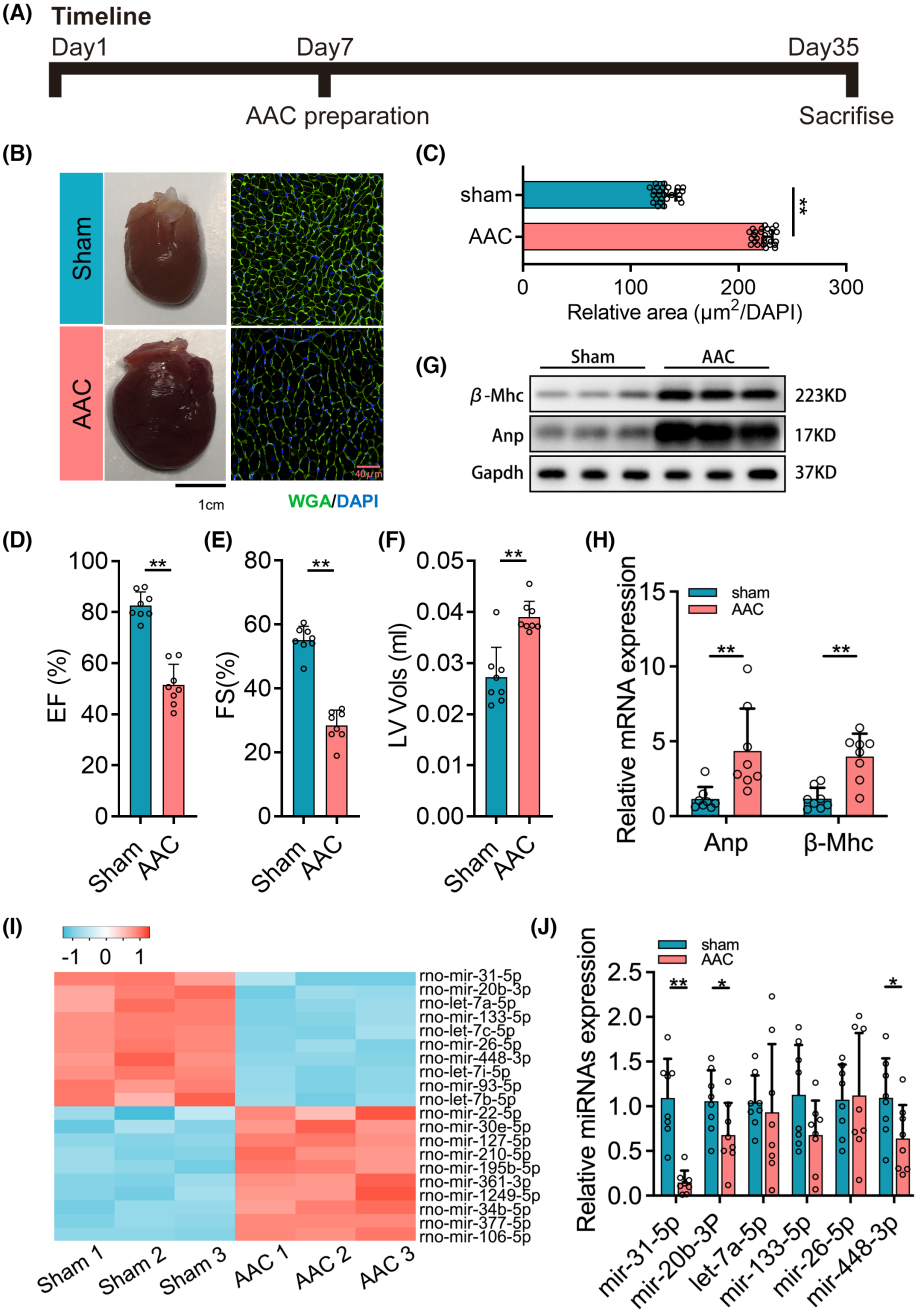
miR‐31‐5p is downregulated in abdominal aorta coarctation (AAC)‐induced myocardial hypertrophy and dysfunction. (A) The timeline of rat AAC experiments (*n* = 8). (B) The representative pictures of the heart and wheat germ agglutinin (WGA) staining of the sham group and AAC group. (C) The relative area of the myocardial cell surface (*n* = 8, 3 fields per sample). (D–F) Echocardiography analyses of cardiac function of sham and AAC groups (*n* = 8). (G, H) The expression of myocardial hypertrophy biomarkers including Anp and β‐Mhc was measured by western blot (*n* = 6) and qPCR (*n* = 8, 3 repeats). (I) Heat map for the top 10 upregulated and downregulated microRNAs in the AAC‐induced group compared with the sham group (*n* = 3). (J) The verification for the altered microRNAs presented in the heat map (*n* = 8, 3 repeats). For two groups, unpaired *t*‐tests were used. And for comparison of more than two groups, one‐way ANOVA analysis was processed, and the significance is expressed as follows: *p < 0.05, **p < 0.01.

Rats underwent surgery the AAC model on Day 7, followed by killing on Day 35 (Figure 1A). The hearts of AAC‐induced rats were larger than those in the sham group; moreover, the cardiomyocytes revealed by WGA staining and the relative area in AAC‐induced heart tissue were increased than the sham group rats (Figure 1B,C). Consequently, the cardiac function was detected by echocardiography, with rats in the AAC group showing decreased EF and FS but increased LV wall thickness (Figure 1D–F). Meanwhile, the protein and mRNA levels of cardiac hypertrophy biomarkers Anp and β‐Mhc were significantly upregulated in AAC rats (Figure 1G,H). The above results indicated a successful early cardiac hypertrophy in rats by AAC induction. Then, we explored the alterations in microRNAs in the induced heart tissue using microRNA microarray assay. The top10 increased or decreased microRNAs were as follows: miR‐31‐5p, mir‐20b‐3p, let‐7a‐5p and so on (Figure 1B,C). For this study, we verified six more microRNAs (shown in Figure 1J) through qPCR testing, which showed a consistent decrease, particularly miR‐31‐5p. Taken together, miR‐31‐5p may play a key role in cardiomyocyte remodelling during cardiac hypertrophy processing.

### 3.2 miR‐31‐5p suppress cardiac hypertrophy in vitro and in vivo

**FIGURE 2:**
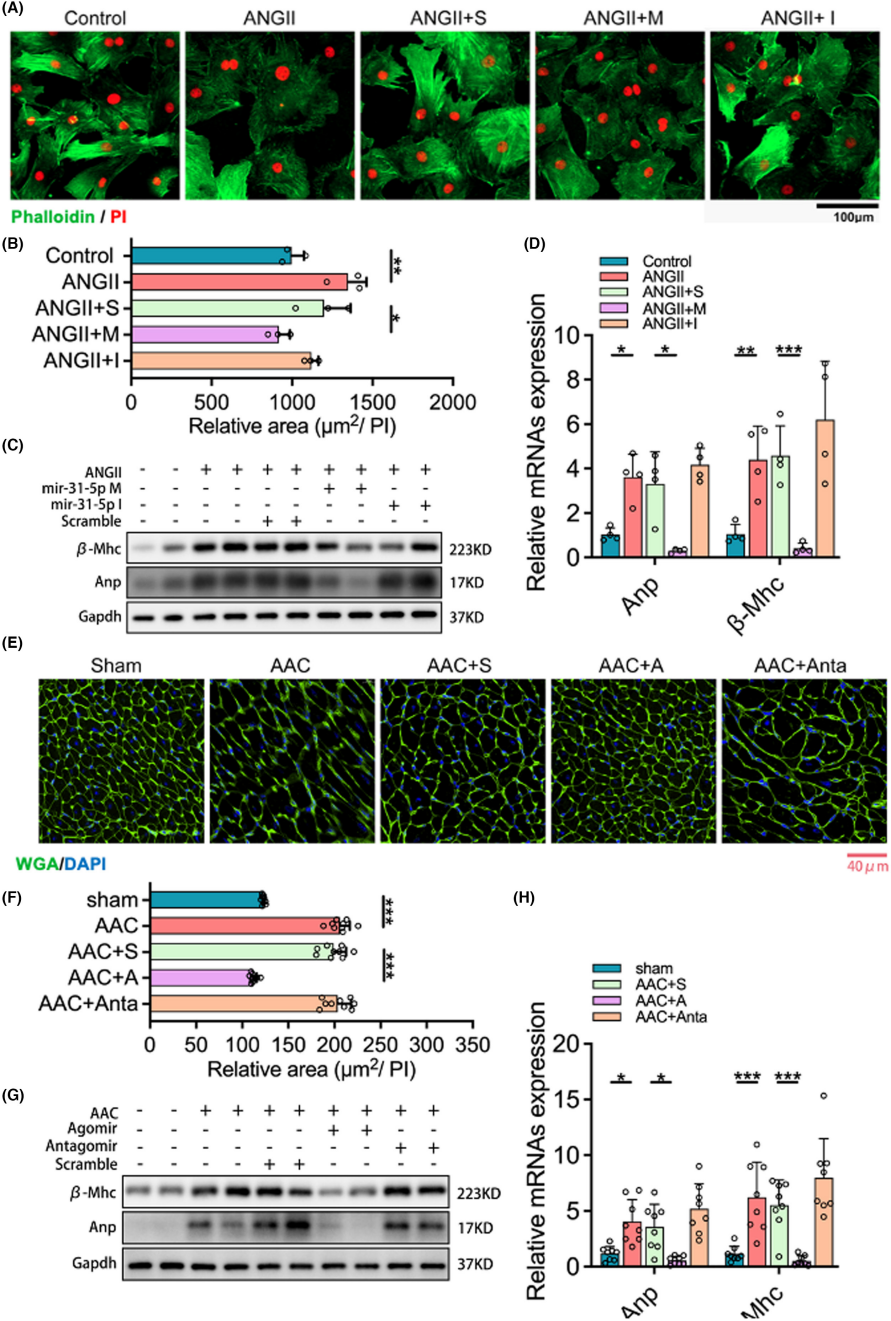
miR‐31‐5p inhibits myocardial hypertrophy in vivo and in vitro. (A, B) The surface areas of neonatal rat cardiomyocytes (NRCMs) induced by ANGII were revealed by cell actin with phalloidin (green) staining and cell nuclear with PI (red) staining. Thereby the relative area was normalized by nuclear numbers (*n* = 8). S, scramble; M/I, miR‐31‐5p mimic or miR‐31‐5p inhibitor. (C, D) The expression of Anp and β‐Mhc in NRCMs was measured by WB (*n* = 4) and qPCR (*n* = 4, 3 repeats). (E, F) The surface of rat myocardial cells is illustrated by wheat germ agglutinin (WGA) staining (green) and 6‐diamidino‐2‐phenylindole (DAPI) (blue) for nucleus, followed by the relative area that was statistically characterized (*n* = 3, 8 fields per sample). S, scramble; A/Anta, miR‐31‐5p agomir or miR‐31‐5p antagomir. (G, H) The expression of Anp and β‐Mhc in rat heart tissue was measured by WB (*n* = 4) and qPCR (*n* = 4, 3 repeats). One‐way ANOVA analysis was processed, and the significance is expressed as follows: *p < 0.05, **p < 0.01, ***p < 0.001.

Because the miR‐31‐5p expression downregulated in AAC‐induced rats significantly, we hypothesized that miR‐31‐5p might play a suppressive role in cardiac hypertrophy. To verify this notion, we performed gain and loss approaches for miR‐31‐5p in vitro and in vivo. First, we verified the expression efficiency of miR‐31‐5p in mimic‐ or inhibitor‐treated NRCMs (Figure S1A). We then transfected miR‐31‐5p mimics, miR‐31‐5p inhibitors or scrambles (as a negative control) in the AngII‐induced NRCM model. Phalloidin staining showed that AngII exposure and transfection with scrambles significantly increased the cell size and relative area of NRCMs compared to the control. While miR‐31‐5p mimics reversed the increase, miR‐31‐5p inhibitors enhanced the increase (Figure 2A,B). Moreover, the mRNA and protein levels of the hypertrophic biomarkers Anp and β‐Mhc were upregulated in AAC rats, which was largely reversed by transfection with miR‐31‐5p mimics. However, transfection with the miR‐31‐5p inhibitor made an upregulation (Figure 2C,D).

Further, the efficiency of miR‐31‐5p expression treated with agomir and antagomir was confirmed in rats (Figure S1B). Rats underwent surgery to induce the AAC model at Day 7; then, miR‐31‐5p Agomir, miR‐31‐5p Antagomir and scrambles were injected twice on Days 10 and 21, respectively (Figure S1C). Echocardiography showed impaired cardiac function, including decreased EF and FS but increased LV wall thickness of AAC rats compared with control rats. There was no significant difference between the AAC and scrambles groups. Compared to the scrambles, miR‐31‐5p agomir reversed the impaired cardiac function while miR‐31‐5p antagomir enhanced that (Figure S1D–F). In AAC rats injected with scrambles, similar to the cells induced with AngII, levels of Anp and β‐Mhc were increased along with an increase in cell size (Figure 2E,H). These results indicated that miR‐31‐5p could suppress cardiac hypertrophy.

### 3.3 miR‐31‐5p targets Nfatc2ip by binding to its 3′‐UTR

**FIGURE 3:**
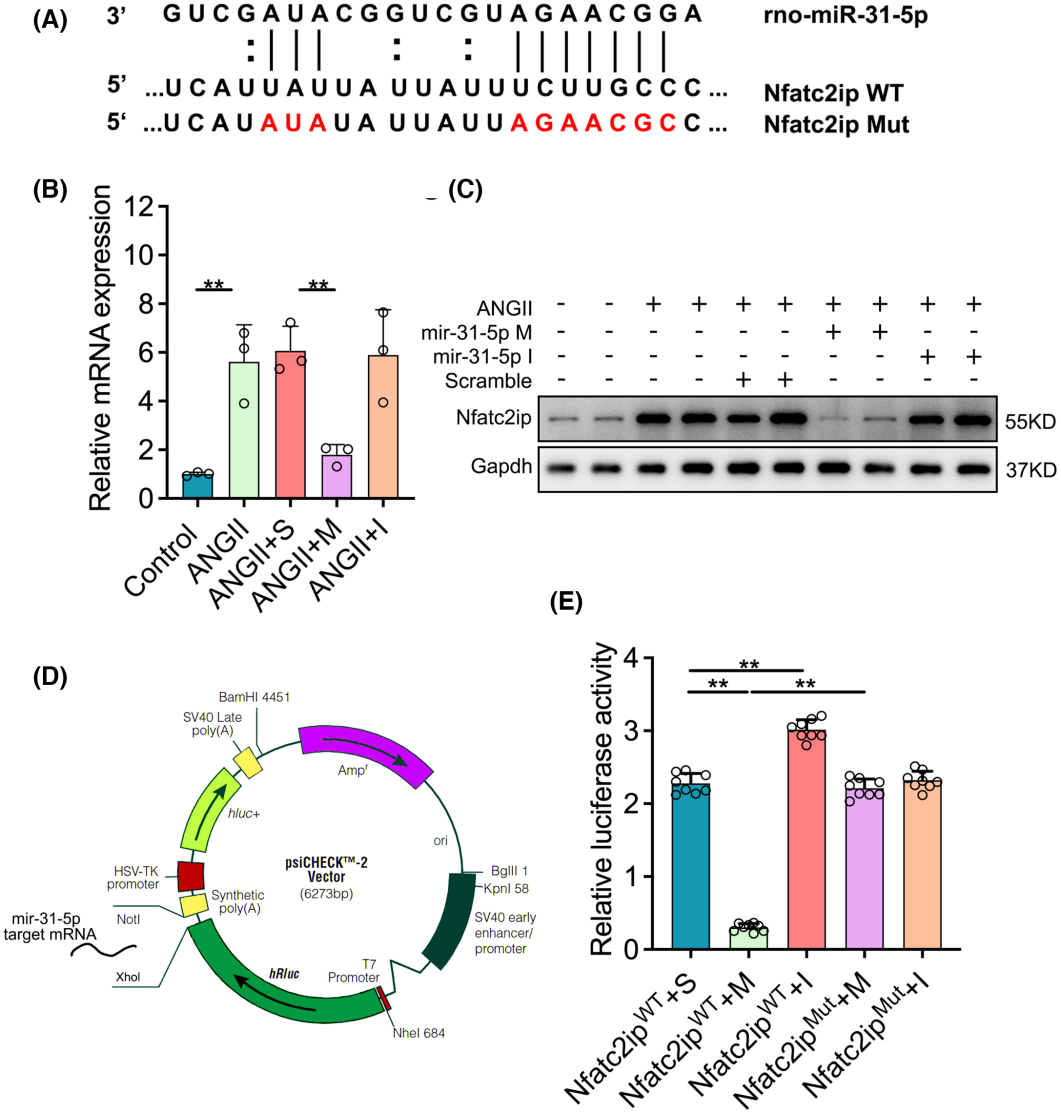
miR‐31‐5p targets Nfatc2ip by binding to its 3′‐UTR. (A, B) The expression of Nfatc2ip in cells was measured by qPCR (*n* = 4, 3 repeats) and WB (*n* = 4). (C) The binding sites of miR‐31‐5p to Nftc2ip 3′UTR (wild type, WT) and the mutant of Nfatc2ip 3′URT (mutant type, MT) were designed to block the binding. (D) The pisCHECK2‐based dual luciferase reporter plasmids were designed with miR‐31‐5p 3′UTR WT/MT inserted after T7 promoted the Rellina luciferase (hRluc) gene, and TK promoted Firefly luciferase (hLuc) as an internal control. (E) Nfatc2ip‐WT or Nfatc2ip MT were co‐transfected with miR‐31‐5p mimic or inhibitor respectively to H9C2 cells. One‐way ANOVA analysis was processed, and the significance is expressed as follows: *p < 0.05, **p < 0.01.

To explore the mechanism of miR‐31‐5p suppressing cardiac hypertrophy, we analysed its potential targets using bioinformatic programmes, including miRbase, microRNA.org and miRcode, predicting that miR‐31‐5p may effectively target Nfatc2ip through binding its 3′‐UTR (Figure 3A). Therefore, we detected the mRNA and protein levels in the NCRMs mentioned above. Nfatc2ip expression was increased in AngII‐induced cells compared to the control group, whereas it was downregulated by miR‐31‐5p mimics and upregulated by miR‐31‐5p inhibitors (Figure 3B,C). To experimentally validate Nfatc2ip as a direct target of miR‐31‐5p, we constructed a luciferase reporter vector containing 3′‐UTR of Nfatc2ip (Nfatc2ip^WT^) and the mutant 3′‐UTR of Nfatc2ip (Nfatc2ip^MT^) (Figure 3A,D). The Nfatc2ip^WT^ and Nfatc2ip^MT^ plasmids were co‐transfected with miR‐31‐5p mimics or inhibitors, respectively, into H9C2 cells. As shown in Figure 3E, miR‐31‐5p strongly inhibited the luciferase activity of Nfatc2ip^WT^, and its inhibitors increased the luciferase activity. Unsurprisingly, no effect was observed with the corresponding mutant construct. The above results demonstrated that Nfatc2ip is the target of miR‐31‐5p.

### 3.4 Nfatc2ip contributes to myocardiocyte hypertrophy

**FIGURE 4:**
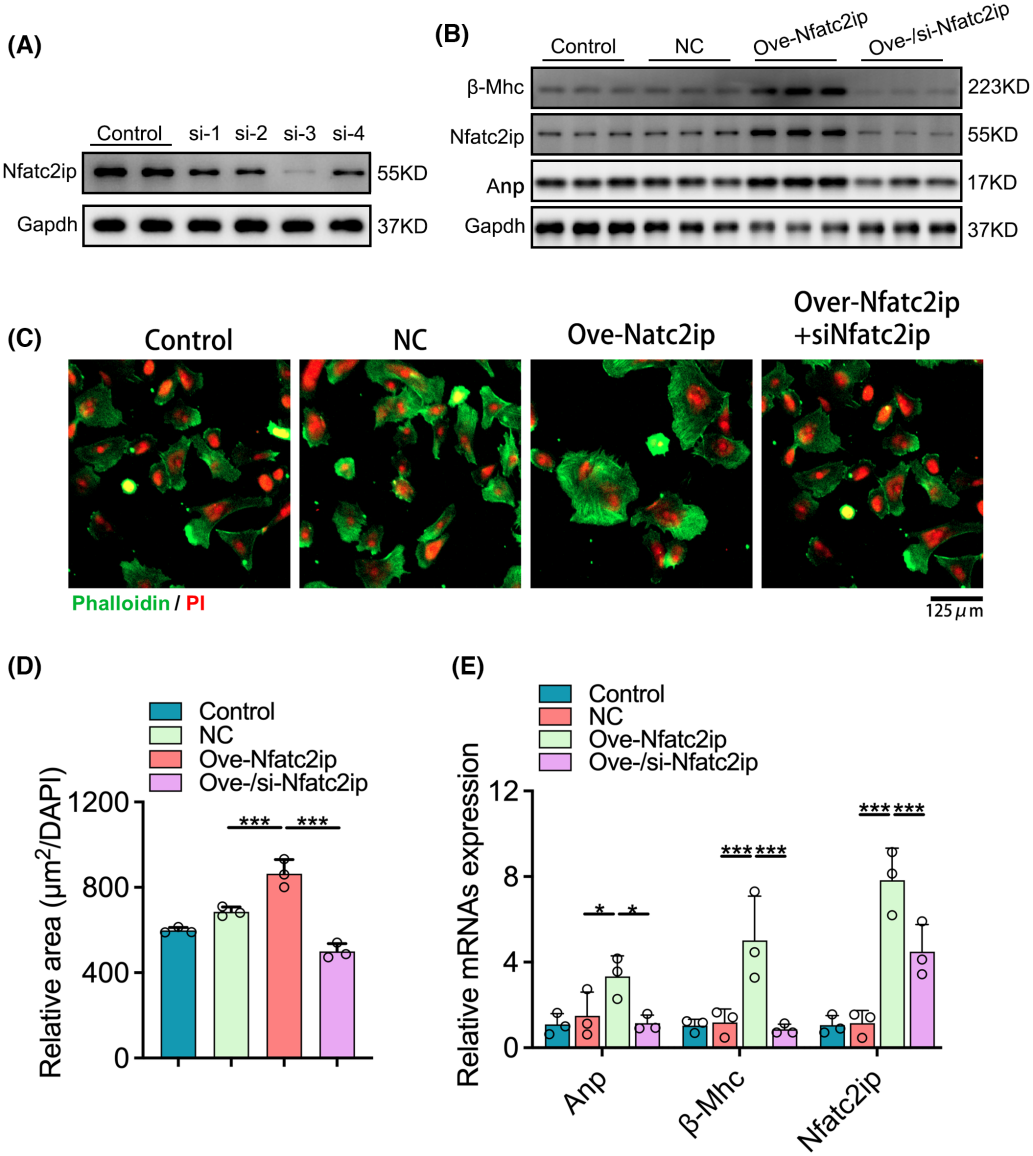
Nfatc2ip is necessary and sufficient for cardiac hypertrophy. (A) The Nfatc2ip inhibition by siRNA fragments s (si1–si4) transferred into H9C2 cells is revealed by WB. (B) The protein level of Anp, β‐Mhc and Nfatc2ip was detected by WB (*n* = 3). NC presents cells infected with vector lentivirus and transfected with scramble fragments. Ove‐Nfatc2ip means cells infected with lentivirus‐based overexpression of Nfatc2ip, and Ove−/si‐Nfatc2ip is defined as infected with Nfatc2ip lentivirus and then transfected with the si‐Nfatc2ip fragment. (C, D) The cell surface area was illustrated with Phalloidin (green), and the relative area was calculated (*n* = 3, 3 fields/ sample). (E) The mRNA level of Anp, β‐Mhc and Nfatc2ip was detected by qPCR (*n* = 3, 3 repeats). One‐way ANOVA was used to analyse the statistics, and the significance is expressed as follows: *p < 0.05, ***p < 0.001.

To determine whether Nfatc2ip is a crucial gene for the hypertrophic remodelling of myocardiocytes, we applied the lentivirus containing the Nfatc2ip gene to make up the Nfatc2ip‐overexpressed H9C2 cell line. si‐RNAs of Nfatc2ip, as a rescue trail, were transfected into cells, showing that the fourth si‐RNA of Nfatc2ip (si‐4) was the most effective (Figure 4A). Cells infected with lentivirus‐containing vector and transfected using scrambles were defined as negative control (NC). The increased cell size (Figure 4B,C) along with hypertrophic gene expression—Anp, β‐Mhc and Nfatc2ip—were remarkably increased in Nfatc2ip‐overexpressed cells (Figure 4D,E), whereas they were rescued by Nfatc2ip siRNA. These results demonstrated that the upregulated Nfatc2ip contributes to cardiac hypertrophy.

### 3.5 miR‐31‐5p inhibits myocardial hypertrophy through blocking Nfatc2ip transcriptional function

**FIGURE 5:**
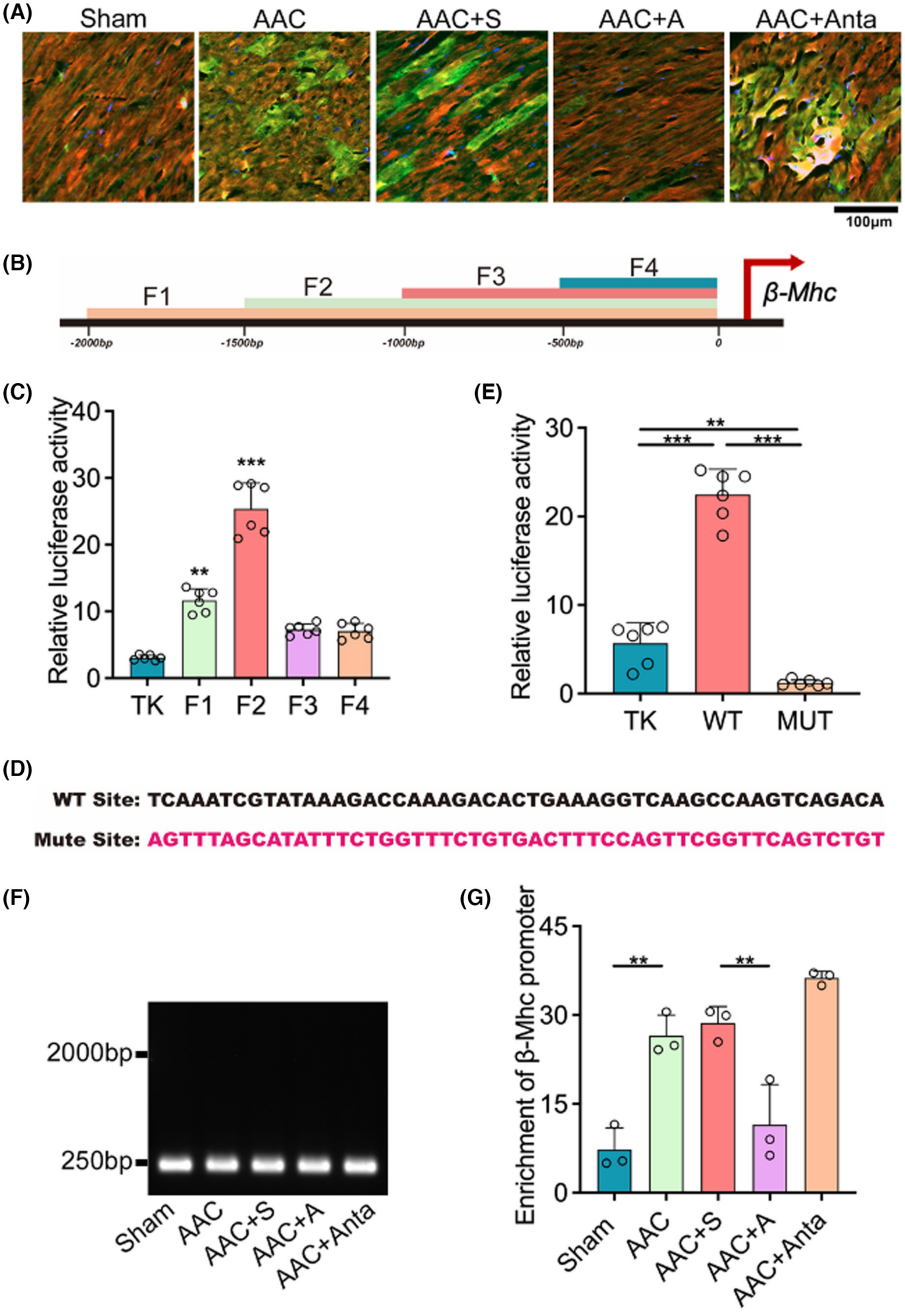
miR‐31‐5p blocks Nfatc2ip transcriptional function ameliorating myocardial hypertrophy. (A) Representative pictures of co‐staining of Nfatc2ip (green) and β‐Mhc (red) in rat heart tissue. The nucleus is stained with DAPI (blue) (*n* = 3). (B) The representative fragments of Nfatc2ip promoter from −500 to −2000 bp. The dual‐luciferase reporter reveals the most responsible fragment of the promoter. (C) The fragments mentioned were inserted into the pGL3‐basic plasmid promoting Luc gene, and a control plasmid expressed Rellina luciferase (hRluc) initiated with TK promoter. They were co‐transfected into H9C2 cells (*n* = 6, 3 replicates). (D) Sequences of core‐promoter (WT) and the related mutant (MT). (E) The transactivational function of WT or MT of core‐promoter revealed by the dual‐luciferase re‐porter (*n* = 6, 3 replicates). (F) Representative picture of the DNA shearing fragments (around 250 bp) with sonication followed by performed chromatin. (G) Chip‐qPCR detection revealed the enrichment of core‐promoter of β‐Mhc infected with Nfatc2ip antibody (*n* = 3). One‐way ANOVA was used for statistical analysis, and the significance is expressed as follows: *p < 0.05, **p < 0.01, ***p < 0.001.

Nfatc2ip, known as a transcriptional factor, belongs to the NFATC family, and has been demonstrated to be involved in the transcription of the hypertrophic‐related genes in the early stage of hypertrophic remodelling of myocardiocytes, which is thought to be an important marker for cardiac hypertrophy.^9^, ^10^ Overexpression of β‐Mhc has been verified to be disadvantageous in cardiac function under severe cardiovascular stress and accelerated LV hypertrophy with isoproterenol stimulation,^21^ suggesting that β‐Mhc may probably be a potential target downstream of Nfatc2ip. To verify this notion, Nfatc2ip and β‐Mhc were co‐stained with immunofluorescence in AAC rats, which were treated with miR‐31‐5p mimics, miR‐31‐5p inhibitors and scrambles. The colocalized cells were increased in AAC and AAC+S group rats compared to the sham group. While miR‐31‐5p agomir significantly decreased the colocalized cells, miR‐31‐5p antagomir increased that compared to AAC+S, suggesting a positive correlation between Nfatc2ip and β‐Mhc (Figure 5A). Always, the 0–2000 bp before the initiation transcriptional site is defined as the gene's promoter. To determine whether Nfatc2ip will transactivate β‐Mhc, we further designed the luciferase plasmids derived from the fragmental promoter of β‐Mhc (Figure 5B), which were co‐transfected with the TK‐derived plasmid as inner control. Figure 5C shows that Fragment 2 had the highest relative luciferase activity among the four fragments, suggesting the co‐promoter (CP) is in the −1000 to −1500 bp range. By bioinformatic programme, the potential CP was predicted, and the mutant sites were designed as shown in Figure 5D. Consequent relative luciferase activity was found to be increased in cells transfected with WT plasmid and decreased with Mut plasmid, which confirmed the CP promoter of β‐Mhc is localized at 1165–1214 bp of β‐Mhc. Moreover, we sheared the DNA from the nuclear protein isolated from rat hearts into the 250‐bp segment, then incubated with a magnetic bead containing the Nfatc2ip antibody, followed by qPCR with primers designed for the CP of β‐Mhc. The results revealed a significant increase in the enrichment of β‐Mhc promoter in AAC, AAC+S and AAC+Anta groups; however, AAC+A remarkably decreased that. These results demonstrated that Nfatc2ip activated transcription of β‐Mhc through binding to the CP of β‐Mhc, and miR‐31‐5p inhibited the β‐Mhc expression by targeting Nfatc2ip which blocked the transcription of β‐Mhc.

## 4 DISCUSSION

This study revealed significantly downregulated miR‐31‐5p in AAC‐induced cardiac hypertrophic rats. Mechanistically, miR‐31‐5p suppresses cardiac hypertrophy in vivo and in vitro by targeting Nfatc2ip. Nfatc2ip overexpression induced myocardial hypertrophy, which is rescued by si‐Nfatc2ip RNA. Moreover, β‐Mhc, known as a biomarker of cardiac hypertrophy and reported to be involved in remodelling myocardiocytes in the early stage of overload pressure‐induced cardiac hypertrophy, was transactivated by Nfatc2ip binding to its core‐promoter. Taken together, our findings showed that miR‐31‐5p was downregulated in AAC‐induced rats, leading to the suppression of Nfatc2ip removed, resulting in upregulated b‐Mhc, which initiates cardiomyocyte remodelling in the early stage of cardiac hypertrophy.

Cardiac hypertrophy is an adaptive response to pressure overload, clinically, which is correlated with an increase in the incidence of cardiovascular disease and is often the initial step in the progression to congestive heart failure. Animal models of cardiac hypertrophy are crucial for researching the pathogenesis and developing therapeutic strategies for preventing cardiac hypertrophy. Many researches have revealed the various mechanisms of hypertrophic remodelling of myocardiocytes. Most of them used an induction duration of more than 6 weeks, while a few focused on the early stage of cardiac hypertrophy. According to Wenjun Dai's work,^20^ we used rats weighing 80–120 g to make up a 4‐week AAC‐induced cardiac hypertrophic model, which are younger rats rather than adults. Our data also showed that 4‐week induction by AAC showed significantly impaired cardiac function, increased heart size and cardiomyocyte size, and increased cardiac hypertrophic biomarkers Anp and b‐Mhc. These results demonstrate that 4‐week AAC‐induced young rats are an appropriate animal model for studying cardiac hypertrophy in the early stage.

Accumulative miRNAs have been characterized mainly by pro‐hypertrophic and anti‐hypertrophic miRNAs. Our microarray assay has shown that miR‐22, miR‐195 and miR‐377 were upregulated, and let‐7 microRNA families, miR‐133 and miR‐26 were downregulated in the cardiac hypertrophy, as observed previously.^7^, ^22^, ^23^, ^24^, ^25^, ^26^ Interestingly, miR‐31‐5p was first found to be significantly downregulated in 4‐week AAC‐induced young rats in our study. miR‐31‐5p has been reported to be involved in cancer and T cells. Moreover, downregulated miR‐31‐5p was reported to promote aortic aneurysm/dissection, which implies miR‐31‐5p plays a role in cardiac‐associated diseases. Although the miR‐31‐5p inhibitor reversed the increased cardiomyocyte (H9C2 cell line) size induced by sh‐LncRNA Tincr, this induction may be caused by many complex factors, which cannot be equated with the induction of overload pressure. Due to the diversity of miRNA targets, the impact on a disease is determined by the combined effects of multiple targets. Our data have demonstrated that miR‐31‐5p mimic or agomir can suppress the hypertrophic cell in vivo and in vitro along with the downregulated protein and mRNA levels of cardiac hypertrophic biomarkers. Together with all these findings, our study first found that miR‐31‐5p is an anti‐hypertrophic miRNA that is downregulated in the early stage of cardiac hypertrophy.

Despite miR‐31‐5p, Nfatc2ip is predicted to be targeted by various microRNAs, and one piece of evidence has proved that miR‐301b‐5p targets Nfatc2ip to regulate inflammation in the liver.^27^ Though studies about Nfatc2ip in cardiac hypertrophy are scarce, Nfatc2ip is significantly upregulated in the third month after ST‐elevation myocardial infarction,^11^ some of which have proposed that Nfatc2ip is involved in common variable immunodeficiency^28^ or calcium channel TRPV6‐mediated breast cancer.^29^ In this study, we verified that miR‐31‐5p targets Nfatc2ip by binding to its 3′‐UTR. Moreover, we first found out that Nfatc2ip is upregulated in the early stage of myocardial hypertrophy, and the overexpression of Nfatc2ip‐induced myocardial hypertrophy is rescued by si‐Nfatc2ip RNA. Our results established a mechanistic link between miR‐31‐5p and hypertrophy, with Nfatc2ip serving as a mediator of the anti‐hypertrophic signal of miR‐31‐5p.

Our another significant finding is that Nfatc2ip promotes the transcriptional activity of β‐Mhc. Mammalian cardiac muscle expresses two genes encoding myosin heavy chains (Mhc), including α‐ and β‐Mhc. Cardiac hypertrophy is accompanied by the remodelling of myocardiocytes, which decreases α‐Mhc and increases β‐Mhc.^30^ β‐Mhc is an essential and multifunctional target of cardiac hypertrophy.^21^ Patients with defective β‐Mhc gene mutation were more vulnerable to cardiac hypertrophy^31^; however, overexpressed β‐Mhc augmented the hypertrophic level.^21^ While found to promote the length of myocardiocytes,^32^ others thought that β‐Mhc is predominantly involved in fibrosis.^33^ Our data first showed elevated β‐Mhc corresponding to decreased miR‐31‐5p and increased Nfatc2ip protein level. The fibrosis in our study has not been evaluated, which may be investigated further. Moreover, recent studies have focused on functional analyses of the β‐Mhc gene promoter,^34^, ^35^, ^36^ suggesting the interplay of *cis* and *trans* factors in the regulation of β‐Mhc. Most of these studies focused on the 0–408 promoter and enhancer element −2900 to −3500 bp.^37^ Our study also suggested an interaction between the promoter of β‐Mhc and the transcriptional factor Nfatc2ip. Through fragmental β‐Mhc promoter‐derived luciferase reporter test, the more effective promoter sequence within −1000 to −1500 bp was determined, followed by mutation, confirming the core‐promoter of β‐Mhc, which was verified by Chip‐qPCR.

**FIGURE 6:**
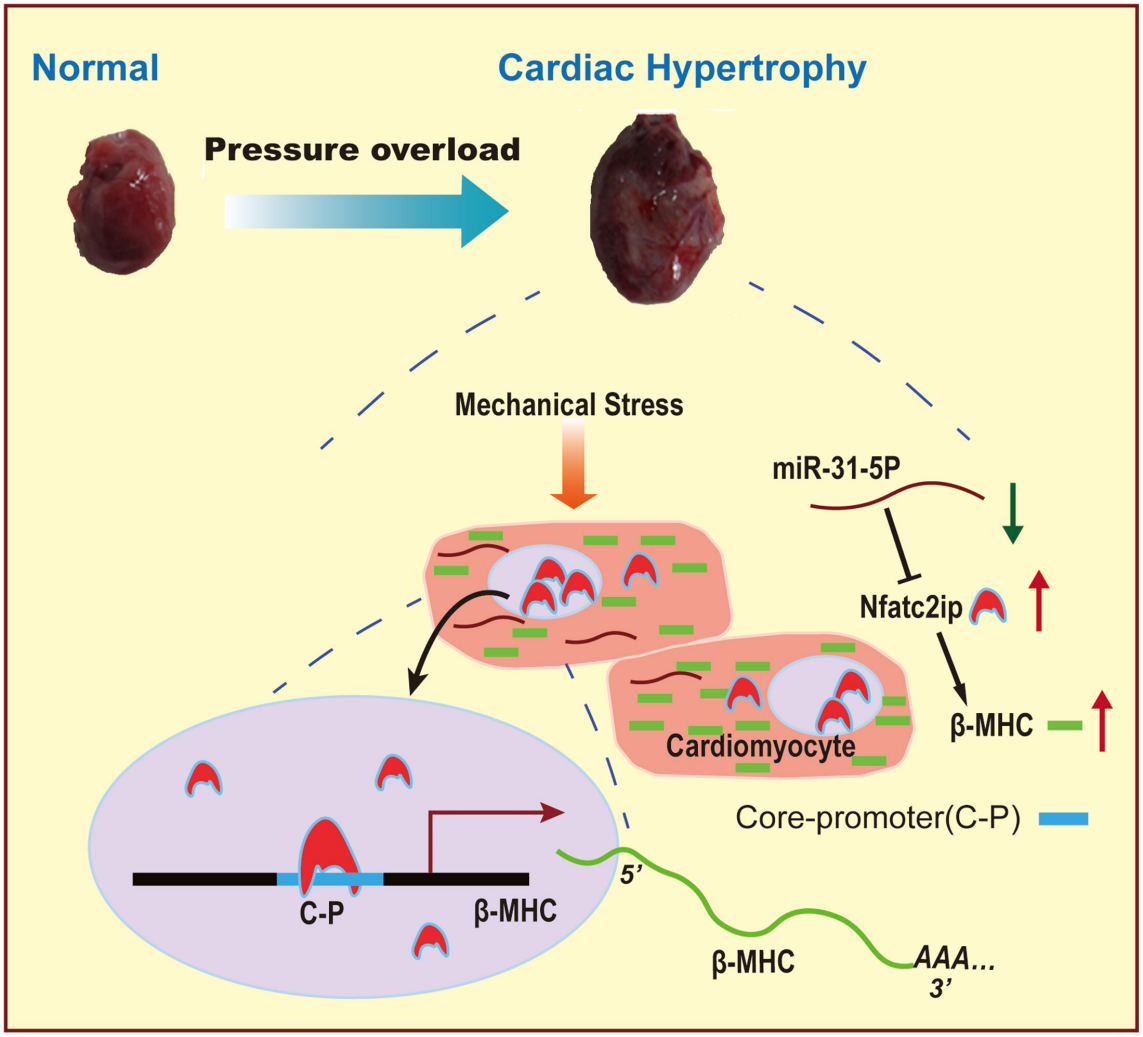
The overview of this study. The pressure overload‐induced mechanical stress on the cardiomyocytes results in downregulated miR‐31‐5p, which inhibits the Nfatc2ip translation through binding to the 3′‐UTR of Nfatc2ip mRNA, followed by more core‐promoters of β‐Mhc leading to upregulated β‐Mhc transcription that accelerate cardiac hypertrophy.

Our data demonstrate that miR‐31‐5p inhibits hypertrophic remodelling in early cardiac hypertrophy. Furthermore, Nfatc2ip, a target of miR‐31‐5p, contributes to myocardiocyte hypertrophy. Moreover, β‐Mhc, transactivated by Nfatc2ip, is blocked by miR‐31‐5p (Figure 6). The study points to a new pathogenic mechanism for microRNA‐associated cardiac hypertrophy, suggesting the miR‐31‐5p/Nfatc2ip/β‐Mhc pathway as a potential target for cardiac hypertrophy.

## AUTHOR CONTRIBUTIONS

**Lamei Zhao:** Conceptualization (equal); data curation (equal); formal analysis (equal); investigation (equal); methodology (equal); project administration (equal); software (equal); visualization (equal); writing – original draft (lead). **Xiaotao Qian:** Data curation (equal); formal analysis (equal); investigation (equal); methodology (lead); software (equal); validation (equal); visualization (equal); writing – original draft (equal). **Zhenxing Ren:** Conceptualization (lead); formal analysis (equal); investigation (equal); methodology (equal); software (equal); visualization (equal); writing – review and editing (equal). **Ailing Wang:** Conceptualization (equal); data curation (equal); formal analysis (supporting); investigation (equal); project administration (equal); resources (equal); supervision (lead); writing – review and editing (lead).

## FUNDING INFORMATION

Not applicable.

## CONFLICT OF INTEREST STATEMENT

The authors declare that they have no competing interests.

## Supporting information


**Figure S1.** Echocardiography analyses of cardiac function. (A, B) The verification of expression efficiency of miR‐31‐5p mimic/inhibitor in cell and agomir/antagomir in rats (*n* = 3, 3 repeats). (C) The timeline of rat abdominal aorta coarctation experiments (*n* = 8). (D–F) Echocardiography analyses of cardiac function. One‐way ANOVA was analysed, and the significance is expressed as follows: **p < 0.01, ***p < 0.001.

## Data Availability

The datasets used and/or analyzed during the current study are available from the corresponding author on reasonable request.
