# Supplementary figures and images for: miR‐31‐5p suppresses myocardial hypertrophy by targeting Nfatc2ip
Source: J Cell Mol Med. 2024 Jun 19;28(12):e18413. doi: 10.1111/jcmm.18413 (PMC11187844; doi:10.1111/jcmm.18413)

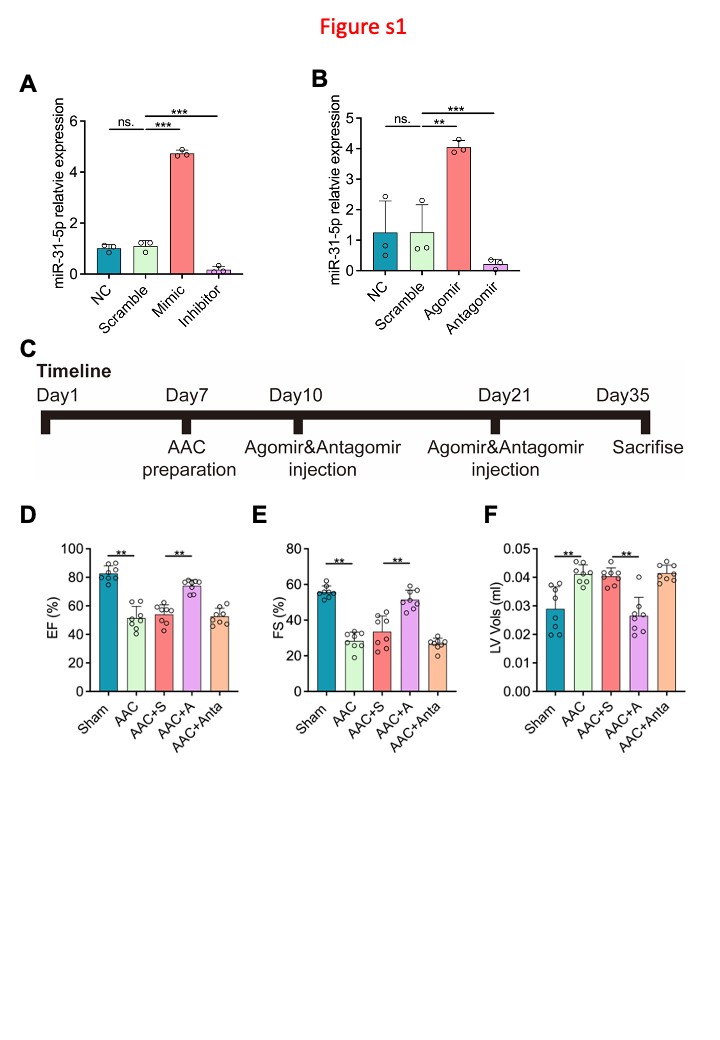

Supplement: Supplementary file 1 — Figure S1. Echocardiography analyses of cardiac function. (A, B) The verification of expression efficiency of miR‐31‐5p mimic/inhibitor in cell and agomir/antagomir in rats (n = 3, 3 repeats). (C) The timeline of rat abdominal aorta coarctation experiments (n = 8). (D–F) Echocardiography analyses of cardiac function. One‐way ANOVA was analysed, and the significance is expressed as follows: **p < 0.01, ***p < 0.001. [file JCMM-28-e18413-s001.tiff]
